# Cerebral Anatomy Detection and Surgical Planning in Patients with Anterior Skull Base Meningiomas Using a Virtual Reality Technique

**DOI:** 10.3390/jcm10040681

**Published:** 2021-02-10

**Authors:** Samer Zawy Alsofy, Makoto Nakamura, Ayman Suleiman, Ioanna Sakellaropoulou, Heinz Welzel Saravia, David Shalamberidze, Asem Salma, Ralf Stroop

**Affiliations:** 1 Department of Medicine, Faculty of Health, Witten/Herdecke University, 58448 Witten, Germany; ralf@stroop.de; 2 Department of Neurosurgery, St. Barbara-Hospital, Academic Hospital of Westfälische Wilhelms-University Münster, 59073 Hamm, Germany; ayman.suleiman@hotmail.com (A.S.); iSakellaropoulou@barbaraklinik.de (I.S.); hwelzel@barbaraklinik.de (H.W.S.); 3 Department of Neurosurgery, Academic Hospital Köln-Merheim, Witten/Herdecke University, 51109 Köln, Germany; NakamuraM@kliniken-koeln.de; 4 Department of Neurosurgery, Klinikum Hochsauerland, Academic Hospital of Westfälische Wilhelms-University Münster, 59821 Arnsberg, Germany; d.shalamberidze@klinikum-hochsauerland.de; 5 Department of Neurosurgery, St. Rita’s Neuroscience Institute, Lima, OH 45801, USA; asem.salma@gmail.com

**Keywords:** cerebral anatomy, meningioma, skull base, surgical planning, three-dimensional reconstruction, tumor resection, virtual reality

## Abstract

Anterior skull base meningiomas represent a wide cohort of tumors with different locations, extensions, configurations, and anatomical relationships. Diagnosis of these tumors and review of their therapies are inseparably connected with cranial imaging. We analyzed the influence of three-dimensional-virtual reality (3D-VR) reconstructions versus conventional computed tomography (CT) and magnetic resonance imaging (MRI) images (two-dimensional (2D) and screen 3D) on the identification of anatomical structures and on the surgical planning in patients with anterior skull base meningiomas. Medical files were retrospectively analyzed regarding patient- and disease-related data. Preoperative 2D-CT and 2D-MRI scans were retrospectively reconstructed to 3D-VR images and visualized via VR software to detect the characteristics of tumors. A questionnaire of experienced neurosurgeons evaluated the influence of the VR visualization technique on identification of tumor morphology and relevant anatomy and on surgical strategy. Thirty patients were included and 600 answer sheets were evaluated. The 3D-VR modality significantly influenced the detection of tumor-related anatomical structures (*p* = 0.002), recommended head positioning (*p* = 0.005), and surgical approach (*p* = 0.03). Therefore, the reconstruction of conventional preoperative 2D scans into 3D images and the spatial and anatomical presentation in VR models enabled greater understanding of anatomy and pathology, and thus influenced operation planning and strategy.

## 1. Introduction

Anterior skull base meningiomas represent a wide cohort of tumors with different locations, extensions, and histotypes [1]. These meningiomas can be challenging tumors that abut the olfactory and optic nerves, compress the frontal lobes, and invade the basal bony structures [2]. Surgical resection is the main treatment option for tumors that are symptomatic and/or growing [3], and can be achieved using several approaches such as pterional, bifrontal, and transbasal [2]. The ideal surgical approach should provide adequate exposure of the tumor, surrounding structures, and dural attachments and should aim to minimize brain retraction and avoid manipulation of vital neurovascular structures [4]. The decision for an approach is made based on tumor size and extension, size of the frontal sinus, anatomical conditions, and surgeon’s preference [5].

Diagnoses of anterior skull base meningiomas and review of their therapies over time are inseparably connected with cranial tomographic imaging. Different conventional radiographic studies are available to delineate the size and morphologic features and to determine anatomical relationships of these tumors, including magnetic resonance imaging (MRI), magnetic resonance angiography (MRA), computed tomography (CT), and computed tomography angiography (CTA) [6]. These modalities result in two-dimensional (2D) images. The ability to generate three-dimensional (3D) images from 2D images might improve the radiographic evaluation, and thus facilitate decisions regarding the appropriate treatment strategy [7].

The 3D reconstructions of imaging modalities, which were originally mainly presented on flat screens, enabled better understanding of spatial and anatomical relationships. In the 1990s, several articles on surgical virtual reality (VR) were published [8]. In recent years, VR technology has become increasingly important in many medical fields, including neurosurgery [9,10]. The current VR visualization technology enables transition from conventional 3D screen images to interactive 3D-VR models. This is associated with many benefits for operation planning, explanation of surgical procedures for patients, medical deduction, and clinical training, such as improved understanding of the detailed anatomy and configuration of cerebral aneurysms [11,12,13,14]. The continuous advances in medical technology and development of portable electronic devices has improved the user friendliness of VR technology for operators, medical students, nursing staff, and other employees integrated into the healthcare system [12].

The concept behind modern VR is the transformation of 3D images into the stereoscopic patient model, using CT and MRI scans to create an exact and accurate representation of the complex anatomy using a cost-effective method, with additional implementation possibilities in procedures such as minimally-invasive and endoscopic surgeries [15,16]. Furthermore, preoperative 3D-VR models have been reported to be in high agreement with intraoperative conditions; the resulting intraoperative “déjà vu” feeling strengthened surgical confidence [17].

In our study, we retrospectively evaluated a cohort of patients who underwent surgical treatment for anterior skull base meningiomas. We intended to answer the question of whether 3D-VR-based visualization of reconstructed preoperative CT and MRI scans would result in a recommended surgical strategy that deviated from the recommended strategy based on conventional interpretation of the same, orthogonal-orientated CT and MRI scans.

## 2. Materials and Methods

The study protocol was approved by the ethics commission of the Medical Faculty, Witten/Herdecke University (Ref-Nr. 201/2018).

### 2.1. Patient Enrolment

In analyzing our hospital information system, we retrospectively identified patients within a 4-year period (2016–2019) who underwent surgical resection of meningioma, and matched the following inclusion criteria: (1) adult age, (2) anterior skull base meningiomas, (3) preoperative reconstructable, thin-slice cranial CT modalities (including CTA) and MRI modalities (including MRA) with axial, sagittal, and coronal views. To obtain a homogeneous patient group, the following exclusion criteria were defined: (1) young age, (2) multimorbid patients, (3) other cranial pathologies, (4) previous craniotomies, (5) recurrent meningiomas, (6) multiple meningiomas.

### 2.2. Data Acquisition and Handling

Data from all included patients were retrospectively analyzed. Patient data were collected from patient files, discharge papers, surgical reports, outpatient reports, and imaging reviews. Patient- and disease-related data, including age, gender, preoperative imaging, tumor location, tumor size, surgical procedure, histopathological findings, perioperative complications (during surgery and within the first two weeks after surgery), and discharge status were collected, analyzed, and evaluated. The extent of surgical resection was determined on the basis of the surgeon’s estimation, as documented in the surgical reports, as well as on the evaluation of postoperative CT and MRI scans.

### 2.3. Neurosurgical Technique

The surgical procedure was performed according to a largely uniform technique [5], as follows. The head was fixed in a three-pin Mayfield headholder after suitable positioning. An incision according to the planned approach (pterional or extended pterional, supraorbital, frontal or bifrontal, combined) was performed. The scalp and galea flap were mobilized and reflected inferiorly and/or laterally. The bone flap was removed with the help of a cranial drill and a craniotome. Under microscope, the dura mater was then opened and the brain gently retracted to locate, prepare, and resect the meningioma. The dura mater was then closed, and the bone flap was reinserted into the cranial defect and fixed to the bone with miniplates and miniscrews.

### 2.4. Virtual Reality Visualization Technique

**Figure 1:**
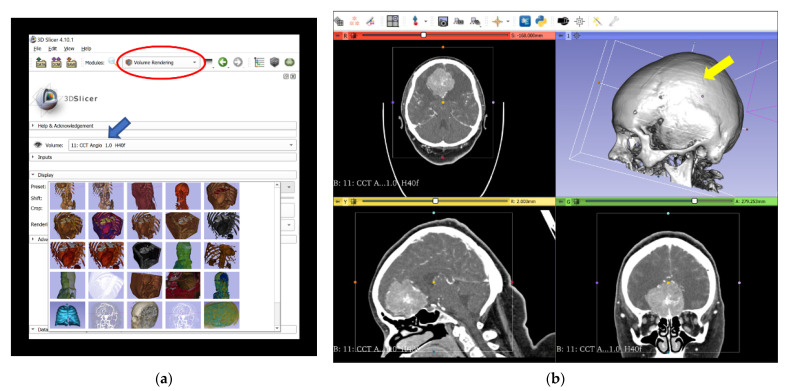

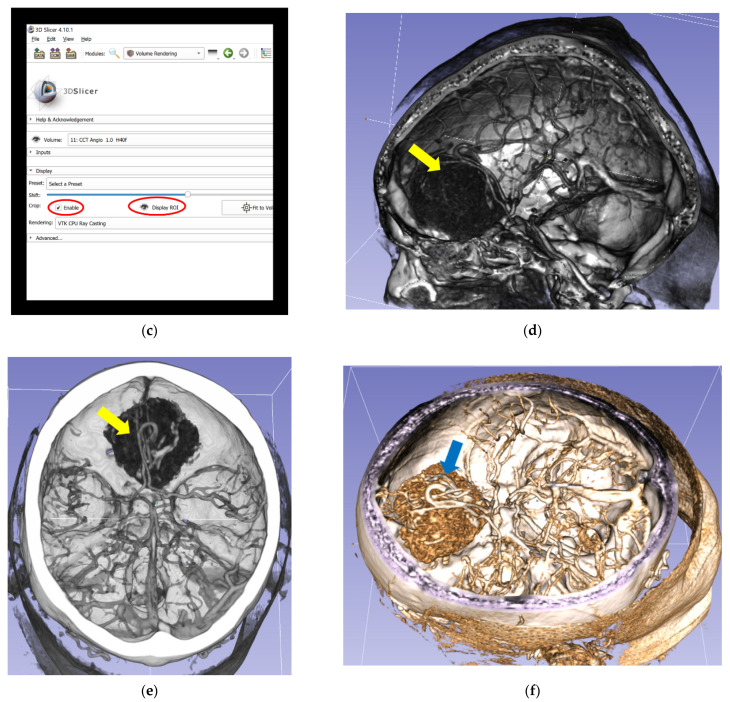
Reconstruction process of 3D-VR images from 2D-CT modalities (including CTA) and completion of the final VR scene in the 3D Slicer. (**a**) Import of original CT and CTA data in an anonymized DICOM format into 3D Slicer software to create a patient-specific database and selection of “CCT Angio-Default” (blue arrow) in the volume rendering window (red circled). (**b**) Performance of 3D-VR reconstruction of a skull (yellow arrow) in the volume rendering window. (**c**) Activation of the ROI function (red circled), which enables visualization of the meningioma and relevant vascular anatomy from different perspectives by partial omission of skull bones. (**d**) Lateral aspect of the meningioma (yellow arrow) and relevant vascular anatomy, simplified by using the ROI function. (**e**) Superior aspect of the meningioma (yellow arrow). (**f**) Superior lateral aspect of the meningioma (blue arrow) with different color displays. 2D, two-dimensional; 3D, three-dimensional; CT, computed tomography; CCT, cranial computed tomography; CTA, computed tomography angiography; DICOM, digital imaging and communications in medicine; ROI, regions of interest; VR, virtual reality.

The digital imaging and communications in medicine (DICOM) files of preoperative 2D-CT and 2D-MRI scans were retrospectively reconstructed to 3D-VR images. We used open-source medical image analysis and visualization software (3D Slicer, Surgical Planning Laboratory, Harvard University, USA) [18], which runs on a VR workstation (main board: Intel Core i7-6800 K (Intel Corporation, Santa Clara, CA, USA); RAM: 16 GB; graphic card: 2 X NVIDIA GTX 1080 (NVIDIA Corporation, Santa Clara, CA, USA)) connected to HTC Vive (HTC Corporation, Xindian District, New Taipei City, Taiwan) goggles, and the SteamVR tracking and controller system (Valve Corporation, Bellevue, WA, USA). The steps of the reconstruction process from 2D-CT modalities, which are the same steps for the reconstruction process from 2D-MRI modalities, are shown in Figure 1. To assess the effectiveness of this method, the duration of the reconstruction process, including completion of the final VR scene, was calculated.

### 2.5. Study Design

**Figure 2:**
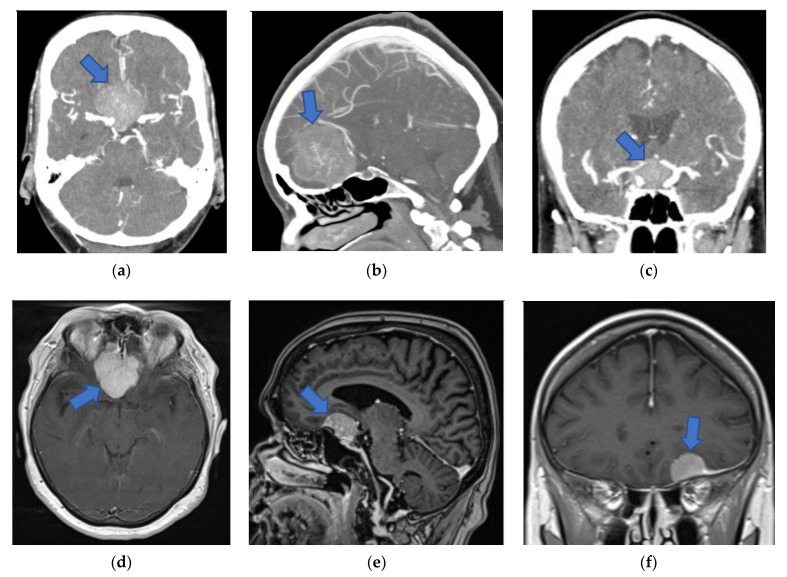

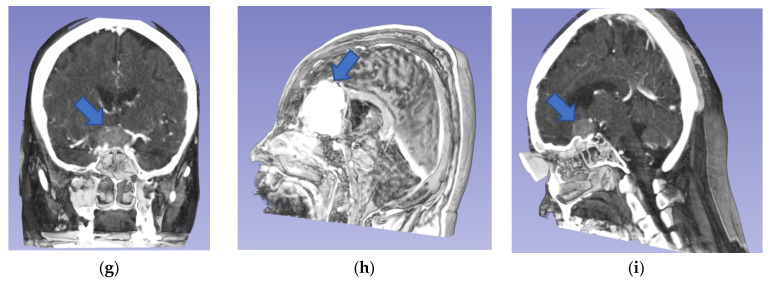
Preoperative 2D and screen 3D images (from CT and MRI modalities) of patients with anterior skull base meningiomas (blue arrows). (**a**) Axial 2D-CTA image presenting planum sphenoidale meningioma. (**b**) Sagittal 2D-CTA image presenting olfactory groove meningioma. (**c**) Coronal 2D-CTA image presenting tuberculum sellae meningioma. (**d**) Axial 2D-MRI image presenting olfactory groove meningioma. (**e**) Sagittal 2D-MRI image presenting anterior clinoidal meningioma. (**f**) Coronal 2D-MRI image presenting frontobasal meningioma. (**g**) Coronal screen 3D-CT image presenting tuberculum sellae meningioma. (**h**) Sagittal screen 3D-MRI image presenting olfactory groove meningioma. (**i**) Sagittal screen 3D-CT image presenting anterior clinoidal meningioma. 2D, two-dimensional; 3D, three-dimensional; CT, computed tomography; CTA, computed tomography angiography; MRI, magnetic resonance imaging.

**Figure 3:**
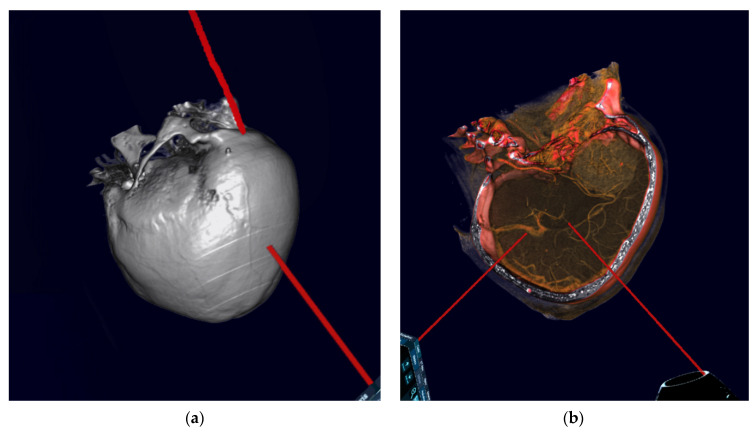
Virtual reality-based head positioning of patient with anterior skull base meningioma. (**a**) Spatial lateral head rotation. (**b**) Representation of the tumor morphology and the anatomical relationships in the same lateral head rotation.

**Figure 4:**
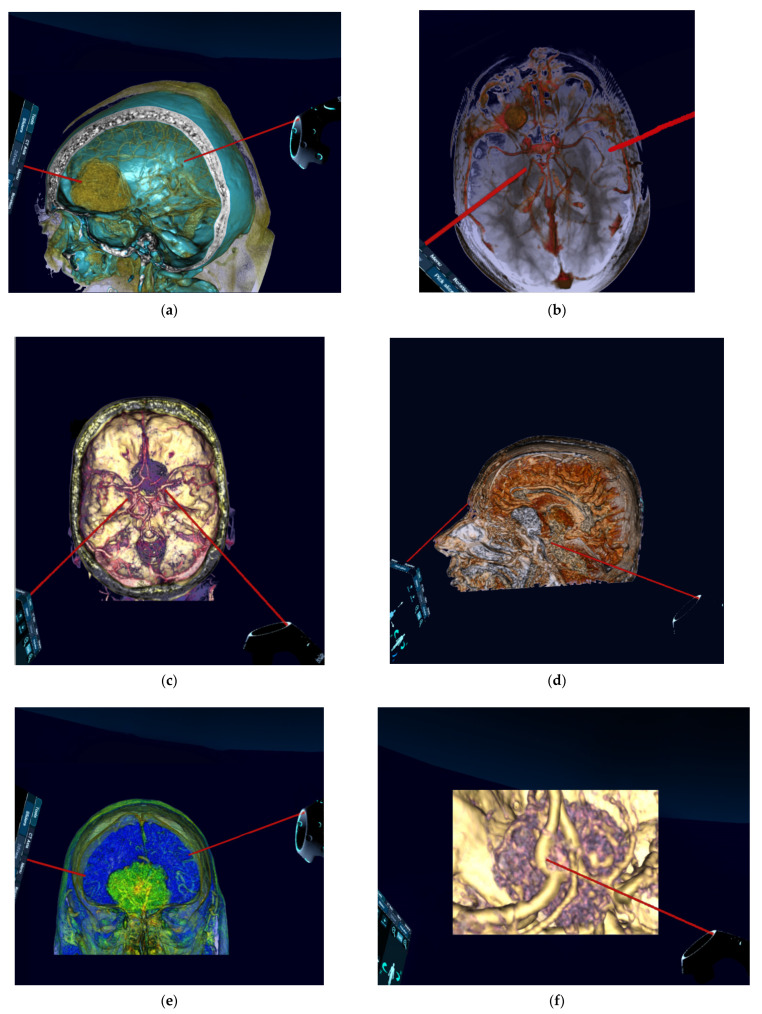

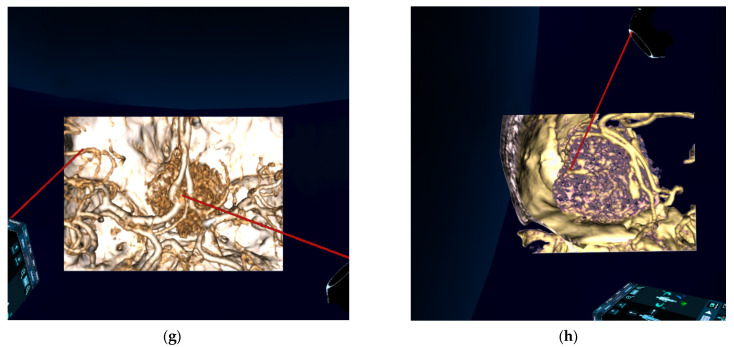
Preoperative reconstructed 3D-virtual reality images (reconstructed from 2D-CT and 2D-MRI) of patients with anterior skull base meningiomas. (**a**) Oblique lateral aspect showing olfactory groove meningioma. (**b**) Superior aspect showing frontobasal meningioma. (**c**) Superior aspect showing planum sphenoidale meningioma. (**d**) Lateral aspect showing anterior clinoidal meningioma. (**e**) Anterior aspect showing olfactory groove meningioma. (**f**) Highly zoomed superior aspect showing planum sphenoidale meningioma. (**g**) Zoomed superior aspect showing planum sphenoidale meningioma. (**h**) Zoomed oblique lateral aspect showing olfactory groove meningioma. 2D, two-dimensional; 3D, three-dimensional CT, computed tomography; MRI, magnetic resonance imaging.

Conventional preoperative 2D and screen 3D images from CT and MRI scans (examples in Figure 2) of all included patients were retrospectively demonstrated to ten experienced, board-certified neurosurgeons. All of them were senior (attending) consultant neurosurgeons with many years of multi-institutional experience in neurosurgery. Their experience was based on their numerous and qualifying operative engagements as primary or assistant surgeons in in skull base surgeries, including skull base meningeoma operations. To reduce any influence on the recommendations given and to avoid bias, the neurosurgeons who performed the operations and completed the reconstructions were excluded. Using a questionnaire, the included surgeons were asked to evaluate the identification of anatomical structures and to determine the preferred patient positioning, head positioning, and surgical approach (Table 1). The reconstructed 3D-VR images (examples in Figure 3 and Figure 4) of the same patients were retrospectively presented to the same neurosurgeons four weeks later, but in a different order to minimize the influence of the first questionnaire on the second. Again, the neurosurgeons were asked to complete the same questionnaire. To avoid influence from the patient- and disease-related data on the image evaluations, these data were not presented. The possible influence of the preoperative reconstructed 3D-VR images compared to the conventional preoperative CT and MRI scans (2D and screen 3D) on detection of anatomical structures and on surgical planning and strategy was evaluated.

Since the questionnaire was retrospective, the questioned neurosurgeons could give answers regarding the preferred surgical strategy that were different to the procedures that were actually carried out in these patients. To verify the confidence of the questionnaire, the intrarater reliability was determined by re-surveying the neurosurgeons 4 weeks later, as each of the ten neurosurgeons could suggest different evaluations for the same image set by repeated questionnaires.

### 2.6. Statistical Analysis

Patient data were collected anonymously. We applied the Fisher-exact test [19] to estimate the statistical probability of a correlation between two variables by measuring the difference between the collected data and expected values, which would be assumed for uncorrelated factors. We assumed a *p*-value < 0.05 to be significant. For age, tumor size, and duration of the reconstruction process, mean ± standard deviation (SD) values were calculated. The intrarater reliability was determined using Cohen’s kappa coefficient [20].

## 3. Results

### 3.1. Patient- and Disease-Related Data

By reviewing our clinical database of all patients that were operated on for skull base meningiomas within a 4-year period and based on the inclusion and exclusion criteria, thirty patients were enrolled in our study. The mean age was 53 ± 7 (range 32–77) years. The anterior clinoidal and the olfactory groove meningiomas were the most frequent tumors (33% and 23%, respectively). The mean maximum tumor size was 4 ± 2 (1–7) cm. Further patient- and disease-related data are summarized in Table 2.

### 3.2. Role of Image Presentation Modality in the Identification of Anatomical Structures and Surgical Planning

The mean duration of the reconstruction process was 9 ± 4 (range 3–16) minutes. Questioning of ten neurosurgeons to evaluate the 30 patients by first displaying conventional CT and MRI images (300 reply sheets) and then presenting reconstructed 3D-VR images (300 reply sheets) resulted in 600 reply sheets. For the determination of the intrarater reliability, substantial agreement was found (kappa values = 0.71 to 0.79), indicating that the neurosurgeons interviewed did adapt to the VR technology.

#### 3.2.1. Impact on Identification of Anatomical Structures

The 3D-VR modality showed a significant advantage in the visualization of the tumor, as well as the surrounding anatomy compared to the conventional CT and MRI scans; 85% of questioned neurosurgeons found the 3D-VR-based anatomical depiction to be sufficient, compared to 74% of neurosurgeons viewing the conventional images (*p* = 0.002) (Table 3).

#### 3.2.2. Impact on Selection of Patient and Head Positioning

The supine position was mostly recommended by the neurosurgeons, independent of image presentation technique (96% using CT and MRI, 94% using 3D-VR). Other positions (semi-supine or lateral) were only recommended in 6% using VR and in 4% using conventional CT and MRI. Thus, the visualization technique showed no influence on the recommended patient positioning (supine/other positions; *p* = 0.27) (Table 3). However, the recommended head positioning was significantly influenced by the image visualization modality (straight or “neutral”/lateral rotation; *p* = 0.009).

#### 3.2.3. Impact on Selection of Surgical Approach

The pterional or the extended pterional approaches were mostly recommended using the 3D-VR image presentation method (36%), while the supraorbital approach was mostly recommended using CT and MRI (42%). The image presentation technique had a significant influence on the selected surgical approach (pterional or extended pterional/supraorbital/frontal or bifrontal/combined; *p* = 0.03) (Table 3).

## 4. Discussion

In our retrospective study to evaluate the impact of the image visualization modality on surgical planning in patients with anterior skull base meningiomas, the way in which sectional images were viewed (i.e., conventional or 3D-VR) significantly influenced the identification of tumor-related anatomical structures and the recommended head position and surgical approach. The neurosurgeons interviewed evaluated the images retrospectively, without previous knowledge of the surgical procedures that were performed on the patients. We did not concentrate on surgical outcomes because our focus was on the retrospective assessment of preoperative imaging diagnostics.

Prior to skull base surgery, conventional radiological methods including CT, MRI, and other imaging data sets are helpful in displaying skull base bone structures and clearly show brain and nerve tissues, blood vessels, other anatomical structures, and tumors. All types of these imaging technologies have their own unique characteristics, advantages, and application scope [6]. However, these imaging modalities mainly provide a 2D structural inspection of surgical anatomy [6,21,22]. Although conventional 3D reconstructions of preoperative 2D images might simplify the anatomical presentation of anterior skull base pathologies, they have limitations regarding the spatial representation, and they do not completely approximate the anatomy realized under the operating microscope at surgery [23]. Additionally, they are mainly presented on flat screens, which are of different and sometimes insufficient sizes and qualities. Therefore, it is useful to integrate an image presentation method such as VR, which combines the advantages of other modalities into one system, with fewer undesired characteristics.

Black reported in a study that the use of 3D reconstruction in a VR format markedly improved the ability to visualize the tumor and its relation to the vascular structures and to the sagittal and other sinuses, enabled better identification of tumor feeders and its proximity to major arteries, and increased the possibility of establishing tumor location and relation to cortex [24]. Similarly, in studies by Oishi et al. and Stadie et al., VR models were especially advantageous in spatial depiction for microsurgery, including patient-specific anatomical information different from normal anatomy, such as tumor invasion and damage to normal tissues [17,25]. Accordingly, compared to conventional 2D-CT, 2D-MRI, and screen 3D images in our study, the 3D-VR modality showed a significant advantage in visualizing the tumor as well as the surrounding anatomy (Table 3).

An explanation for these results could be that the VR-based observation of the same image modalities allowed a completely free perspective of the anatomical structures from all directions. The surgeon can “step” into the images and gain different insights into the anatomy and explore the different structures, while having the feeling of being part of the VR environment [26]. According to our experience, this technique provides a much more intuitive understanding of the present situs, and even more of the underlying pathology. The VR technique shows the anatomy with a higher magnification and more detail than seen in common radiologic images. VR-based visualization helps to improve radiological evaluation, since limiting factors such as suboptimal background illumination, reflective glare, and visual disturbances can be eliminated, and the object can be focused in front of the goggles [12].

Moreover, the use of VR technology is one of the most promising tools for surgical planning in skull base tumors, including meningiomas [6]. Yashino et al. and Kin et al. reported that VR models can provide the same field of operation and can thus support selection of the most appropriate surgical approach [27,28]. This contributes to improvement of the precision of surgical planning and to reduction of surgical invasiveness and can be beneficial in skull base surgery [9,17,29]. Mert et al. and Tang et al. described that the complex spatial relationships between a tumor and surrounding tissues is difficult to inspect from a 2D sectional image and can be significantly better observed in detail using 3D-VR models [30,31]. Thus, the VR technique is a powerful method that could influence the selection of surgical procedure [24]. This is in compliance with the findings in our study, where the pterional or the extended pterional approaches and thus the lateral head rotation were more selected using the VR visualization modality (Table 3). The small vascular structures adherent or adjacent to the tumor, the complex relationship between tumor and cranial nerves, the degree of tumor infiltration in the skull base, and other anatomical details might be not well visualized in conventional CT and MRI, and thus could be underestimated. The spatial and high-resolution representation of these anatomical structures using the VR technique might explain the increased recommendation of the pterional/extended pterional approach, which might give more possibility for operative control in the case of complex surgical anatomy.

An explanation for this result could be that the view on conventional images does not correspond to the direction of the view on the operative site. The neurosurgeons need to look at 2D images to create mental, spatial, 3D reconstructions of the tumor, tumor-related anatomy, and skull. This process could be difficult and stressful and differs greatly among neurosurgeons. Moreover, due to limitations of mental reconstruction abilities, information might be lost or not precisely processed [26]. The transformation of conventional preoperative 2D images into 3D-VR images, through specific software and technical equipment, simplifies this process. With frequent application, VR technology can perform fast 3D reconstructions of CT, MRI, and other imaging data sets [6]. Furthermore, the 3D-VR models can facilitate not only the spatial reconstruction of tumors, but also the tumors’ relationship to the skull base and vascular structures, as well as to other superficial anatomical landmarks. The neurosurgeons can scale, segment, fuse, and perform other reconstructed imaging tasks [6]. They can freely rotate and position the patient and the head in virtual space. They can also enlarge the head and the vascular structures to the maximum size and navigate along the appropriate corridors and along the vessels and from one structure to another. Using various VR-based applications and possibilities, 3D-VR images can enable a very close resemblance to real operative anatomy and thus provide almost realistic visual feedback for surgery [32]. These possibilities that VR technology enables are limited in 2D images and normal screen 3D reconstructions; therefore 3D-VR image presentation plays an important role in the choice of the head position and surgical approach.

With regard to patient positioning, the adequate positioning and the trajectory adjustment are important key factors for successful resection of most of the anterior skull base meningiomas [4]. Our study showed that retrospective selection was not influenced by the method of viewing the preoperative images (conventional or 3D-VR). The surgical resection of these tumors is mostly carried out using a pterional or extended pterional, supraorbital, or frontal approach. This means that the majority of these tumors can be well approached with the patient in the supine position, without the need for more complicated and time-consuming lateral or semi-supine positionings. This may explain the higher (albeit non-significant) choice of the supine position, independent of image presentation modality (Table 3).

The reconstruction process of 3D-VR models and the finalization of VR scene could be relatively time-demanding at the beginning. However, with practice and after several reconstructions, this process would become routine and necessitate less time. According to our experience, it is worth investing time in order to depict complex anatomy, such as skull base meningiomas, in a three-dimensional, interactive environment. Moreover, the advantages that VR technology offers, including the clear spatial representation of anatomy, could be a valuable tool for employees integrated into the healthcare system [11,12,13,14]. Residents in their neurosurgical training as well as neurosurgeons at the beginning of their professional career with little experience in demanding operations, such as skull base meningiomas, could benefit from this technique. Experienced neurosurgeons could be supported by the surgical planning using VR techniques. Additional tasks, such as medical deduction of nursing staff and medical students as well as operation explanations for patients, could also be simplified by this promising technology.

### Study Limitations and Further Prospects

VR plays an increasing role in many scientific neurosurgical studies, but does not yet represent a routine application. The VR technique can support improved orientation toward anatomical relations, but may also tempt surgeons to neglect the complexity of approaches that leads to a different access strategy. The study is retrospective and therefore there is likely some selection bias as in the selection of the preferred patient position. To what extent the 3D-VR image data presentation can lead not only to a change in the surgical strategy, but also to a favorable change in surgical complication rates or patient outcomes, can only be answered by a prospective, multicenter study with standardized outcome measures. In such a study, the clinical and radiological evaluations can be blinded and a robust analysis performed. VR is still dependent on the quality of input data. At the same time, however, with the possible clinical applications of 3D-VR technology and its consecutive relevance for the surgical strategy, technical and procedural quality requirements and standards must also be defined for the technical equipment and software algorithms. This could improve the quality of VR visualization technology, clarify the real borders between VR and other imaging studies, and better demonstrate its promising features in comparison to other modalities.

## 5. Conclusions

In our retrospective study in patients with anterior skull base meningiomas, the reconstruction of conventional preoperative 2D scans into 3D images to enable spatial presentation in VR models did not influence existing and established patient positioning methods. However, the VR technique could allow better detection of tumor-related anatomical structures and influence the selection of head positioning and surgical approaches and thus be an important part of surgical planning and strategy. The retrospective form of the study and the small number of patients are drawbacks that can be overcome by a prospective, multicenter study with standardized outcome measures and blinded imaging and clinical evaluation.

## Figures and Tables

**Table 1:** Questionnaire regarding the anatomical structure detection as well as the recommended surgical strategy for patients with anterior skull base meningiomas, using conventional CT and MRI modalities (2D and screen 3D) or 3D-VR presentations.

Surgeon’s Name:
Patient-ID:
1. How is the identification of anatomical structures according to the presented images? □ Sufficient □ Not sufficient
2. Which type of patient position would you choose for the surgical treatment according to the presented images? □ Supine position □ Other positions
3. Which type of head position would you choose for the surgical treatment according to the presented images? □ Straight “neutral” □ Lateral rotation
4. Which approach would you choose for the surgical treatment according to the presented images? □ Pterional or extended pterional □ Supraorbital □ Frontal or bifrontal □ Combined

2D, two-dimensional; 3D, three-dimensional; CT, computed tomography; MRI, magnetic resonance imaging; VR, virtual reality.

**Table 2:** Patient- and disease-related data of included patients with anterior skull base meningiomas. One or more complications per patient were possible.

Characteristics	*n* (%), Unless Otherwise Stated
age (years), mean ± SD (min–max)	53 ± 7 (32–77)
gender:	
male	11 (37)
female	19 (63)
preoperative imaging:	
MRI/MRA	30 (100)
CT/CTA	30 (100)
DSA	3 (10)
tumor location:	
olfactory groove	7 (23)
tuberculum sellae	5 (17)
anterior clinoidal	10 (33)
planum sphenoidale	3 (10)
frontobasal	5 (17)
maximum tumor size (cm), mean ± SD (min–max)	4 ± 2 (1–7)
surgical procedure:	
total resection (Simpson I, II, III)	25 (83)
partial resection	5 (17)
histopathological findings:	
WHO grade I	27 (90)
WHO grade II	3 (10)
WHO grade III	0 (0)
perioperative complications:	
infection	1 (3)
infarction	1 (3)
secondary bleeding	1 (3)
sensomotoric deficits	1 (3)
pseudomeningocele	3 (10)
diabetes insipidus	2 (7)
deep vein thrombosis	1 (3)
discharge status:	
no new symptoms	28 (93)
new neurological symptoms	2 (7)

CT, computed tomography; CTA, computed tomography angiography; DSA, digital subtraction angiography, MRA, magnetic resonance angiography; MRI, magnetic resonance imaging; SD, standard deviation.

**Table 3:** Anatomical structure detection and recommended surgical strategy * after presenting conventional CT and MRI scans (2D and screen 3D) or reconstructed 3D-VR images, evaluated using Fisher’s exact test, assuming a *p*-value <0.05 to be significant.

Anatomical Detections and Recommendations, *n* (%)	2D and Screen 3D (*n* = 300)	3D-VR (*n* = 300)	*p*-Value
anatomical structure detection:			
sufficient	223 (74)	254 (85)	0.002
not sufficient	77 (26)	46 (15)	(significant)
recommended patient positioning:			
supine position	288 (96)	281 (94)	0.27
other positions (semi-supine or lateral)	12 (4)	19 (6)	(not significant)
recommended head positioning:			
straight “neutral”	219 (73)	188 (63)	0.009
lateral rotation	81 (27)	112 (37)	(significant)
recommended surgical approach:			
pterional or extended pterional	77 (25)	107 (36)	
supraorbital	126 (42)	104 (35)	0.03
frontal or bifrontal	89 (30)	77 (25)	(significant)
combined	8 (3)	12 (4)	

* Based on the questionnaire in Table 1. 2D, two-dimensional; 3D, three-dimensional; CT, computed tomography; MRI, magnetic resonance imaging; VR, virtual reality.

## Data Availability

Data are contained within the article.
